# Feasibility and Preliminary Results of a Standardized Stair Climbing Test to Evaluate Cardiorespiratory Fitness in Children and Adolescents in a Non-Clinical Setting: The “Hand Aufs Herz” Study

**DOI:** 10.3390/children12080993

**Published:** 2025-07-28

**Authors:** Federico Morassutti Vitale, Jennifer Wieprecht, Maren Baethmann, Delphina Gomes, Anja Tengler, Roxana Riley, Samar Shamas, Marcel Müller, Guido Mandilaras, Simone Katrin Manai, Maria Jaros, Nikolaus Alexander Haas, Meike Schrader

**Affiliations:** 1 Department of Pediatric Cardiology and Intensive Care, University Hospital, LMU Munich, 81377 Munich, Germany; federico.morassutti@med.uni-muenchen.de (F.M.V.); jennifer.wieprecht@med.uni-muenchen.de (J.W.); maren.baethmann@tum.de (M.B.); delphina.gomes@med.uni-muenchen.de (D.G.); anja.tengler@med.uni-muenchen.de (A.T.); roxana.riley@med.uni-muenchen.de (R.R.); g.mandilaras@klinikum-stuttgart.de (G.M.); simone.dold@med.uni-muenchen.de (S.K.M.); maria.jaros@med.uni-muenchen.de (M.J.);; 2 Institute for Medical Information Processing Biometry and Epidemiology, University Hospital, LMU Munich, 81377 Munich, Germany; samar.shamas@ibe.med.uni-muenchen.de (S.S.); mmueller@ibe.med.uni-muenchen.de (M.M.)

**Keywords:** cardiorespiratory fitness, children and adolescents, stair climbing test, pediatric preventive health, cardiovascular prevention, screening, non-clinical setting

## Abstract

**Background/Objectives**: Cardiorespiratory fitness (CRF) is of great interest in children and adolescents. Due to the limited availability of cardiopulmonary exercise testing, simple and reliable alternatives are needed. A stair climbing test (SCT) for the assessment of CRF developed at the Department of Pediatric Cardiology of the LMU University Hospital in Munich showed a strong correlation with VO_2_max. The aim of this study is to prove its feasibility in a non-clinical setting and to analyse its results in a larger study population. **Methods**: During the “Hand aufs Herz” study, a comprehensive cardiovascular examination was carried out on 922 pupils and siblings (13.2 ± 7.8 years) at a high school in Bavaria. The SCT was performed to evaluate CRF: participants had to run up and down a total of four floors (14.8 m) as quickly as possible without skipping steps or holding on to the banister. Absolute time has been normalized over the standard height of 12 m to allow comparisons with different settings. An SCT Index was calculated to adjust results to the different weights of participants and the exact height of the staircase. **Results**: The SCT proved to be easily feasible and safe in non-clinical contexts. Out of 922 participants, 13 (1.4%) were not able to perform the test, and 3 (0.3%) had to interrupt it following fatigue or stumbling. A total of 827 participants aged from 9 to 17 years (13.1 ± 2.1 years, 45.8% girls) had a mean absolute SCT time of 53.4 ± 6.2 s and 43.3 ± 5.1 s when normalized over 12 m. **Conclusions**: The SCT represents a simple, cost- and time-saving test that allows a rapid and solid assessment of cardiorespiratory fitness in children and adolescents. We could demonstrate that it is safe and feasible in non-clinical contexts. Its short duration and universal applicability are valuable advantages that could facilitate the establishment of a repetitive cardiovascular screening in the pediatric population, particularly in outpatient departments or settings with low-resource systems.

## 1. Introduction

Cardiovascular diseases (CVDs) remain, to this day, the leading cause of death worldwide. It is perhaps less known that the pathogenesis of CVD starts already in childhood and adolescence with pathological vascular changes and subclinical organ damage [1,2,3,4]. In addition, childhood and adolescence are a crucial period where daily habits are formed, which continue to have a huge impact on the cardiovascular risk profile later in life. Especially, a sedentary lifestyle is an important modifiable risk factor for CVD that can be counteracted through an increase in physical activity. Studies have shown that levels of physical activity in European youth remain low and are even decreasing [5,6]. Far from improving only cardiovascular health, physical activity has been shown to benefit muscular and motor development, skeletal strength, cognitive development, and academic achievement, as well as improving self-confidence and mental health [7]. Nonetheless, the objective assessment of physical activity is rather a difficult task because of the lack of a gold standard measurement. Today, self-administered and interview-based questionnaires aim at obtaining the frequency, duration, and type of different physical activities, but suffer from many response biases [8].

Physical fitness has been defined as one’s ability to perform daily activities and physical tasks with optimal performance, endurance, and strength [9]. Its different characterizing components are both health- and skill-related and include muscular strength, endurance, agility, and flexibility. The most studied health-related component is cardiorespiratory or aerobic fitness (CRF). CRF is described as the capacity of the circulatory and respiratory systems to provide skeletal muscle mitochondria with the oxygen needed for energy production to perform physical activity [10]. In the past decades, cardiorespiratory fitness has emerged as a powerful independent risk factor for cardiovascular disease [11,12,13]. Different studies have linked lower CRF and muscular strength in adolescence with higher all-cause mortality, as well as higher cardiovascular- and cancer-specific mortality later in life [14,15,16]. Interestingly, low CRF has also been shown to be a stronger predictor of cardiovascular mortality and morbidity than high BMI. The Henry Ford FIT Study found that physically fit individuals with high BMI (termed “fit but fat”) had a lower all-cause mortality than their unfit peers. In addition, a so-called “obesity paradox” was found in the cohort of the physically unfit, where individuals with higher BMI had a lower all-cause mortality than their slimmer peers [17]. Other studies also showed that obese and overweight individuals with good CRF had a lower cardiovascular risk profile than their unfit peers with lower BMI [17,18,19]. These facts highlight the need for a practical method to evaluate CRF in the pediatric and general population. The golden standard measurement of CRF in the pediatric population is cardiopulmonary exercise testing (CPET) through treadmill or bicycle spiroergometry. The main parameter describing maximal aerobic capacity is maximal oxygen uptake (VO_2_max), which is measured during maximal incremental or ramp exercise testing. However, CPET requires a considerable amount of time and personnel, as well as expensive machinery that is available only in dedicated clinics. Garcia-Rio stated in 2015 that 26% of patients in whom CPET is indicated do not perform the test, mainly because of a lack of availability, even in clinical settings [20]. Furthermore, both treadmill and bicycle spiroergometry are harder to execute in children. Smaller children cannot be set on a bike or may not be accustomed to cycling, whereas additional safety measures are needed on a treadmill. In both cases, performances are prone to be limited by fear or lack of motivation [21]. For these reasons, several submaximal fitness tests, like the 6 Minutes Walking Test (6MWT), the 20-Meter Shuttle Run Test, or different stair climbing tests have been developed to allow a simple and effective estimation of maximal aerobic capacity [22,23,24].

Climbing the stairs is a complex task that involves multiple organ systems and demands considerable muscular recruitment and oxygen consumption. Moreover, quite the opposite of spiroergometry, it is a most familiar and universal task. For these reasons, several stair climbing tests (SCTs) have been developed in different fields and with very different purposes [25,26]. Several different protocols, ranging from single-step to time- or height-limited, or speed measuring SCT, have been developed to predict VO_2_max in healthy adults. An SCT protocolled at our department in 2022 has shown a very strong correlation with VO_2_max measured by treadmill CPET (r = 0.93) in a cohort of healthy children, young adults, and patients with congenital heart disease. In comparison, the 6MWT showed only a moderate correlation with VO_2_max (r = 0.47) [27].

The present study aims to prove the feasibility of the protocolled SCT in a non-clinical context and analyse its results in a larger paediatric study population in order to assess CRF in children and evaluate their cardiovascular risk profile. In the future, normative reference values will be developed, and correlation to other parameters like arm-strength, assessed via the Hand-Grip Test, will be studied.

## 2. Materials and Methods

### 2.1. Study Design

This prospective cross-sectional study was carried out from April to July 2024 as part of the “Hand aufs Herz” (Hand on Heart) Study of the Department for Pediatric Cardiology of the Ludwig-Maximilians-University Hospital in Munich. Under this ongoing, preventive care project, a comprehensive cardiovascular examination is carried out on pupils in schools of the greater Munich metropolitan area. Ethical approval was granted on 15 April 2024 by the Ethics Committee of the Ludwig-Maximilians-University (Project number 24-0147).

### 2.2. Participants

The project was first presented in every class with a short presentation and informative flyers. Applications were then collected after a parents’ evening. Registration was only possible with a written consent form, signed by both caretakers and the participants themselves. 922 pupils and siblings, aged from 6 to 25 years, took part in this study. To assess the feasibility of the SCT, the whole study population was considered. To ensure a critical number for statistical analysis of the results of the SCT, age limits were subsequently set at from 9 to 17 years. The participants who were not able to perform or conclude the SCT were also excluded. Finally, 827 participants were considered for analysis.

### 2.3. Study Procedure

Participants underwent a comprehensive set of cardiovascular examinations, including physical examination, ECG, echocardiography, and a detailed questionnaire about medical and family history, physical activity, and nutrition habits, as well as risk behaviour. To assess CRF, the Stair Climbing Test was performed following the protocol developed at our department [27]. Participants had to run, as quickly as possible, up and down a flight of stairs four times without skipping steps or holding on to the banister. The minimum height to achieve near-maximal effort was set at 12 m in the protocol, which is a rough equivalent of four floors [28]. In our setting, the total height was 14.8 m. Time was recorded using a stopwatch; there was no upper time limit. The absolute time (SCTt [s]) needed to complete the test was later normalized to the standard height of 12 m, to allow comparisons of results in different setups. SCTt/12 m = (SCTt [s] × 12 m)/staircase height [m]. Heart rate (HR), respiratory rate (RR), and oxygen saturation were measured at rest and immediately after the test. A Massimo Rad-G pulsoxymeter was used to measure HR and oxygen saturation. RR was measured by counting the breathing rate over 30 s and multiplying by two. In addition, peak expiratory flow (PEF) and forced expiratory volume in the first second (FEV1) were measured at rest and 5 min following the test, using a Vitalograph-Asma 1 peak-flow-meter (Vitalograph GmbH, Hamburg, Germany). The main purpose was to exclude relevant airway obstruction either before or after the test. Participants were motivated to give their best and achieve maximum performance by the person conducting the test, following a standard set of encouraging phrases. At first, all measurements were carried out by the person conducting the test. Later, some volunteering pupils were trained in the use of the Massimo pulsoxymeter and the measurement of RR and provided valuable help.

The SCT Index was later calculated to adjust results to different weights of participants and the exact height of the staircase [27].

SCT-Index = ((body weight × staircase height)/SCTt)

### 2.4. Statistical Analysis

A tailored domain of the RedCap^®^ software (Version 14.6.7) was used to store the data. Statistical analyses were then performed using IBM SPSS Statistics^®^ 29.0.2.0. Population characteristics were described with Mean and Standard deviation. Given the large study sample, a normal distribution was assumed based on the central limit theorem and confirmed by a Kolgorov–Smirnoff Normality test. A value of *p* < 0.05 was set for significance.

## 3. Results

### 3.1. Study Population

A total of 922 participants aged from 6 to 25 years took part in this study, 496 were male (53.8%) and 426 were female (46.2%). A total of 827 pupils aged between 9 and 17 years who completed the SCT were included in the analyses. The mean (SD) age was 13.1 (2.1) years. Boys amounted to 54.2% and girls to 45.8%. The mean BMI (SD) was 19.4 (3.6) kg/m^2^. The mean (SD) absolute time to complete the SCT (SCTt) was 53.4 (6.2) s. The mean time normalized over 12 m (SCTt/12 m) was 43.3 (5.1) s. The mean SCT Index was 14.7 (4.8) [(kg × m)/s]. Boys had a slightly lower heart rate than girls both at rest and after exertion (boys 81.8 (13.4) bpm, girls 86.6 (13.1) bpm, *p* < 0.001), but no significant sex-specific difference was found in the mean heart rate difference before and after testing (Δ HR), which was 73.6 (15.7) bpm (*p* = 0.34). No significant sex differences were found in respiratory rate at rest (*p* = 0.022) and after exertion (*p* = 0.323), although boys showed a slightly lower increase in RR (*p* = 0.019). All characteristics are shown in Table 1.

### 3.2. Feasibility of the Stair Climbing Test (SCT)

Out of the total 922 participants, 13 participants (1.4%), 4 male (0.8%) and 9 female (2.1%), were not able to perform the stair climbing test (SCT) on the day of the examination. The main reasons were leg injuries, mostly of the knee and foot, that occurred prior to testing. Second came fatigue, in the context of recovery from an illness. Three participants (all female, 0.3% of all participants) had to interrupt the SCT, following exhaustion, leg pain, and a stumbling episode with a light ankle sprain. Other diverse stumbling episodes were observed, where the participants sustained no injuries and resumed the test immediately, leading to no interruption. The light ankle sprain was the only injury sustained during testing (0.01%).

### 3.3. Sex and Age Differences

**Figure 1:**
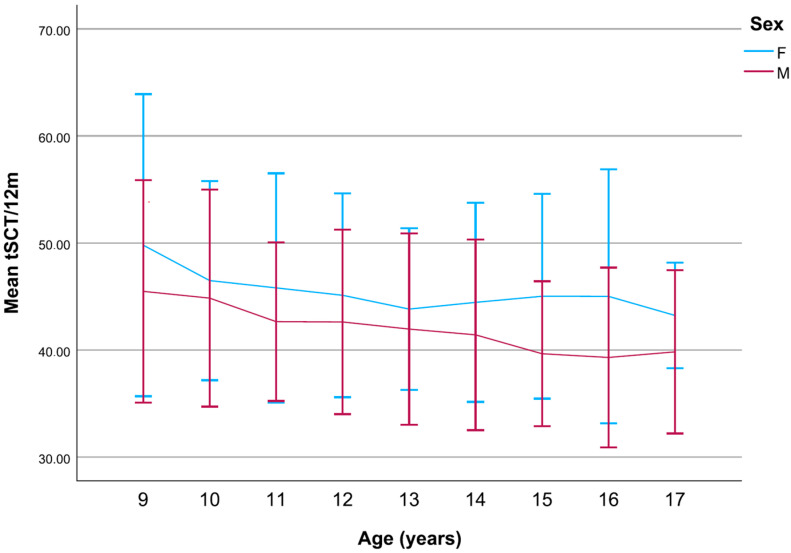
The linear progression of the mean SCTt/12 m in girls and boys compared. The near parallel decrease in mean SCTt (s) between boys and girls opens sharply around the ages of 13 and 14, as boys become faster (*p* < 0.001).

Boys took an average of 51.4 s to complete the SCT, while girls took 55.7 s (a 4.3 s difference in the mean SCTt). Boys were faster than girls in all age groups (*p* < 0.001). Normalized for the standard height of 12 m, the mean times (SCTt/12 m) were 41.7 (4.5) s for boys, and 45.2 (5.0) s for girls. The mean SCTt difference was smallest at the age of 10 (2.0 s) and 13 years (2.3 s) and biggest at the age of 16 (7.0 s) (Figure 1).

**Figure 2:**
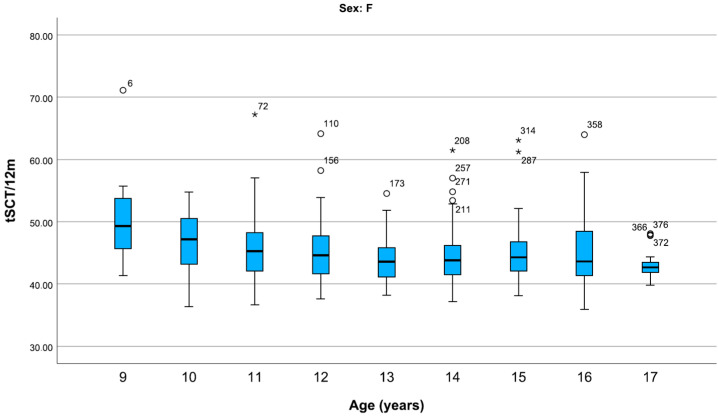
Representation of centiles of the SCTt/12 m (s) in girls, divided by age. For girls, the median SCTt/12 m decreases with age but stabilizes during adolescence. Data are shown in box plots with medians and interquartile ranges. Circles and asterisks represent mild and extreme outliers.

For girls, mean SCTt/12 m fell from the ages of 9 to 13 years but increased in 14- to 16-year-olds, before falling decisively at the age of 17 (Figure 2).

**Figure 3:**
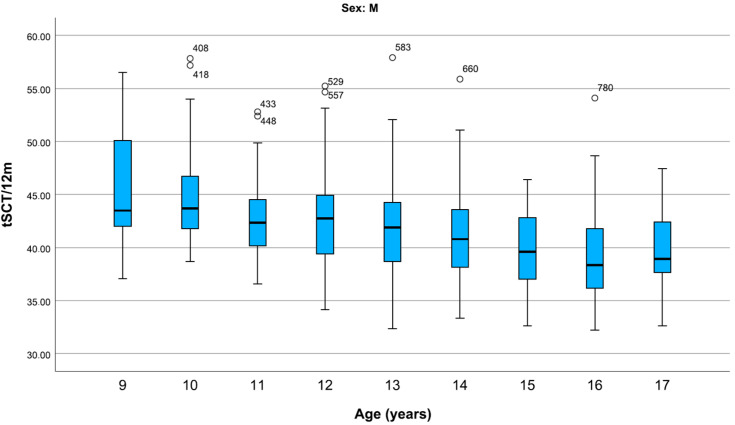
Representation of centiles of the SCTt/12 m (s) in boys, divided by age. For boys, median SCTt/12 m also decreases with age, decreasing more steeply from 13 to 16 years and increasing at 17 years. Data are shown in box plots with medians and interquartile ranges. Circles represent mild outliers.

In boys, the mean SCTt/12 m fell steadily from the age of 9 to 14 years, before falling more steeply from the age of 14 to 16. The 17-year-old boys were slower than the 16-year-olds (Figure 3).

Table 2 shows the percentile distribution of the stair climbing test times for the 827 children and adolescents examined. The test results will be evaluated in future on the basis of the percentiles.

**Figure 4:**
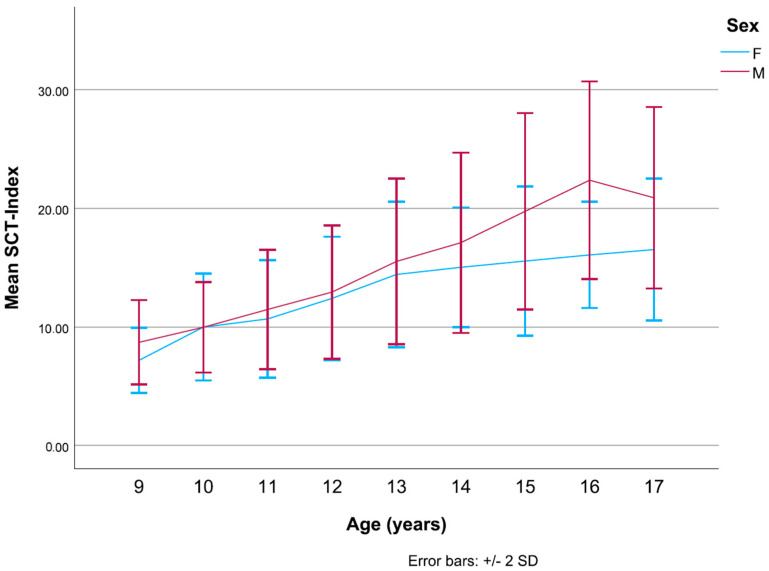
Linear progression of the mean SCT-Index in girls and boys compared. Boys and girls show remarkably similar trends in mean SCT-Index until the age of 13 (*p* < 0.001).

The SCT-Index ((body weight × staircase height)/SCTt) has been calculated to adjust results to the body weight of participants and the exact staircase height. It is inversely related to SCTt, i.e., faster participants have a higher SCT-Index. A significant sex difference was found for the SCT-Index (*p* < 0.001) (Figure 4).

**Figure 5:**
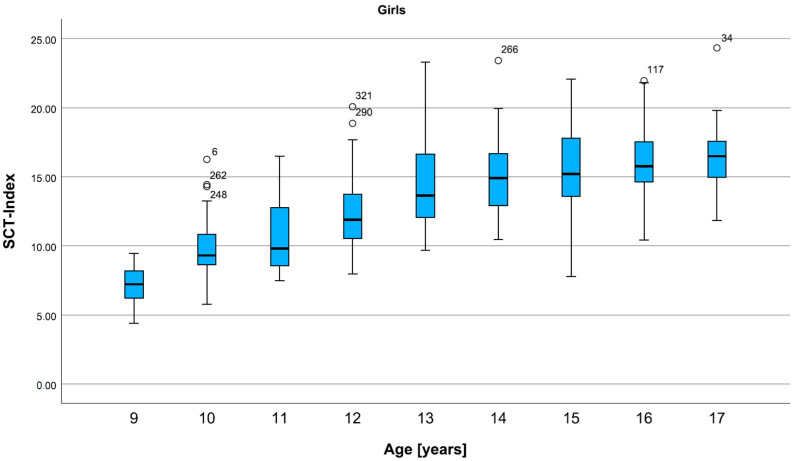
Percentiles for the SCT Index in all ages, for girls. The SCT-Index in girls increases sharply in late childhood and continues to slowly increase in adolescence. Data are shown in box plots with medians and interquartile ranges. Circles represent mild outliers.

For girls, the SCT Index shows a marked increase from 9 to 13 years, then increases slowly, appearing to reach a plateau (Figure 5).

**Figure 6:**
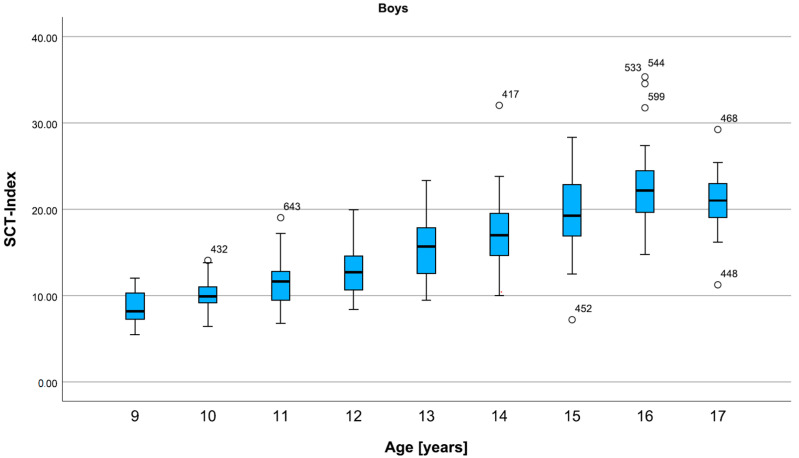
Percentiles for the SCT Index in all ages, for boys. The SCT-Index in boys increases slowly in late childhood and steeply in adolescence. A decrease can be seen in 17-year-olds. Data are shown in box plots with medians and interquartile ranges. Circles represent mild outliers.

For boys, the development is moderate until the age of 13, then steeper until the age of 16. At the age of 17, the SCT-Index is lower than in 16-year-olds (Figure 6).

## 4. Discussion

The results of our study show that the stair climbing test is safe and easily feasible in a non-clinical context. Out of 922 children and adolescents who took part in this study, only 13 (1.4%) were unable to perform the SCT on the day of examination. Out of 909 pupils who carried out the test, only 3 (0.03%) were not able to finish it, and none sustained a serious injury.

In line with other large population studies of cardiorespiratory fitness in children and adolescents, boys consistently outperformed girls in the different age groups and showed larger age-specific differences [29]. Interestingly, once results were adjusted for weight and staircase height, as in the case of the SCT-Index, the mean differences appeared minimal from 9 to 13 years. At 10 years, both girls and boys had a mean SCT-Index of 10.0. This may be related to the fact that girls undergo pubertal physical growth before boys. Differences widen considerably from 13 to 16 when boys, in their turn, experience the physical growth and muscular development typical of puberty. In boys, a decrease in fitness can be observed towards the end of adolescence. This trend coincides with the observed tendency of adolescents to diminish participation in physical activities as educational or professional life changes occur. More studies would be needed to determine how physical activity patterns evolve from adolescence into early adulthood.

### 4.1. Exercise Testing in Children

It has been consistently shown that lower levels of physical activity correlate notably with overweight and obesity, but also with higher markers of CVD, such as higher blood pressure, higher levels of cholesterol, and lower levels of high-density lipoprotein cholesterol [30,31]. In contrast to self-reported physical activity levels, which can vary greatly with time, CRF represents an objective health indicator that can be tracked over time and compared across populations [32].

CPET is also the gold standard assessment of CRF in children, but, not only is its availability restricted, it also presents a lot of limitations, especially in younger children (4–6 years) [21]. Most children-adapted protocols of CPET have a duration of 6–12 min, with the recommendation of keeping it as short as possible to avoid premature muscle fatigue or loss of attention and motivation. Furthermore, the interpretation of standard CPET parameters is difficult in the pediatric population. Half of the children do not reach an oxygen uptake plateau, which defines maximal oxygen uptake (VO_2_max). Instead, peak oxygen uptake is measured (VO_2_peak) [21]. Useful guidelines for pediatric exercise testing can be found in a statement on Clinical Stress Testing in the Pediatric Age Group from the American Heart Association in March 2006 [33].

Our SCT is immediately understandable by children and short in duration (mean SCTt was 54 s, no test exceeded a duration of 90 s). In children, the ludic aspect is important to ensure collaboration and motivation. For this reason, an outdoor CPET with portable devices is currently being studied [34]. Nonetheless, it would continue to involve the use of very expensive machinery with limited availability, especially in non-clinical and low-resource settings.

SCT is a constant work-rate rather than an incremental work-rate test, like CPET [35]. This suggests that, also in the case of our SCT, maximal effort is hard to objectify. Previous research also suggests that submaximal exercise testing may be more reliable in identifying low levels of cardiorespiratory fitness than in recognizing and assessing the fittest part of the population, because very fit individuals may not feel called upon to perform to their highest level [6].

### 4.2. Stair Climbing Tests

Many SCTs have been developed in different fields. In thoracic surgery, SCT served mainly as a tool to predict postoperative outcomes or perioperative complications [20,22]. In the fields of neuromuscular diseases and orthopaedics, SCTs have been used as a marker of functional mobility and disease progression [36]. However, they all differed greatly in methodology [37]. Cataneo et al. developed a fixed-altitude SCT protocol, which would allow the use of the variable time to calculate power and allow the prediction of VO_2_max in adults. Previous studies had identified a height of 12 m as a useful cut-off needed to elicit sufficient effort [28]. Interestingly, the variable time showed a stronger correlation with VO_2_max than power (r = −0.707; *p* ≤ 0.005). This study also highlighted the importance of motivation in reaching near-maximal efforts [37]. Numerous step tests have also been developed to evaluate CRF. The Chester step test has been shown to valuably predict VO_2_max in adults. The test models incremental exercise testing by increasing the stepping rate on a platform of ca. 30 cm, aiming to drive test subjects to exhaustion in 8–12 min [24]. An adaptation of the test has been studied for the prediction of peak oxygen uptake in lean and obese children [38]. However, its total duration is considerably longer, and, so far, it has only been validated in clinical settings.

### 4.3. Limitations

Stair-climbing is a complex task that involves all dimensions of physical fitness, including muscular strength, agility, and coordination. By observation, it was clear that pupils who were more motorically coordinated performed better than their uncoordinated peers, who might have similar levels of CRF. A study found children engaging in multisport to be significantly more coordinated than peers who did not do sports or practiced only swimming [39]. In addition, many participants mentioned the fear of falling as a major restraining factor, especially during stair descent. This aspect allows the recklessness shown by some participants, especially males, to influence test results. Overall, these factors constitute a source of variability of test results, whose role must be explored in future test runs. The sources of variability arising from the test procedure itself should be minimised using standard protocols and technician training, as in the case of the standard sets of encouraging phrases. The other factors must be taken into consideration when interpreting the test results.

Our previous work groups have found that the SCT-Index had a very strong correlation to VO_2_max. However, the correlation to VO_2_max/kg was only moderate, suggesting that physical performance in children could not be simply related to weight [27]. Also, other studies have criticized the adjustment and indexing of VO_2_ on weight, as many variables such as age, somatic growth, and sexual maturity provide complex interactions [40]. Bhammar et al. stated, in 2019, that, without consideration of the effect of fat mass, there could only be an underestimation of CRF in children with obesity, leading to a clinical diagnosis of deconditioning and resulting in unrealistic training goals [41]. Nonetheless, we feel that excessive complexity could compromise the versatility of our SCT. Rather than providing exact predictions of VO_2_max, its greatest value could be to reliably assess CRF by classifying performances in age- and sex-specific intervals, which correspond to lower or higher fitness levels. Personal performances could be then easily tracked in time to follow physical activity adjustments or disease progression, in the case of children with congenital heart disease.

Our study mostly included children older than 8–9 years, with a few exceptions. Further studies would be needed to verify the compatibility of our SCT protocol with younger children or propose adaptations such as stair height. More analyses are needed to determine the extent to which the results of our test correlate with the results of CPET and to define age- and sex-specific normative reference values. In addition, correlations of SCTt and SCT-Index with BMI, muscular strength assessed via Hand-Grip-Test, and reported levels of physical activity will be studied.

## 5. Conclusions

The Stair Climbing Test represents a simple, cost- and time-saving test that allows a rapid and solid assessment of cardiorespiratory fitness in children and adolescents. We could demonstrate that it is safe and feasible in non-clinical contexts. In addition, the normalization of the time needed to complete the test over a standard height of 12 m allows a practical comparison of the results in different setups. Its short duration and universal applicability are valuable advantages that could facilitate the establishment of a repetitive cardiovascular screening in the pediatric population, particularly in outpatient departments and settings with low-resource systems. However, further longitudinal validation studies are needed to strengthen our findings and confirm the predictive value of our SCT in non-clinical contexts.

## Figures and Tables

**Table 1:** Baseline characteristics of the study population.

	All Pupils	Male	Female
Number of pupils, N	827 (100%)	448 (54.2%)	379 (45.8%)
Age, years	13.1 (2.1)	13.1 (2.1)	13.1 (2.1)
Height, cm	162.2 (13.2)	164.3 (14.8)	159.6 (10.4)
Weight, kg	52.0 (15.1)	53.8 (17.0)	50.0 (12.4)
BMI, kg/m^2^	19.4 (3.6)	19.4 (3.8)	19.4 (3.4)
SCT Time, s	53.4 (6.2)	51.4 (5.6)	55.7 (6.2)
SCT Time/12 m, s	43.3 (5.1)	41.7 (4.5)	45.2 (5.0)
SCT Index	14.7 (4.8)	15.7 (5.4)	13.4 (3.6)
HR before the SCT (bpm)	84.0 (13.5)	81.8 (13.4)	86.6 (13.1)
HR after the SCT (bpm)	157.6 (15.3)	155.2 (15.9)	160.4 (13.9)
Δ HR (bpm)	73.6 (15.7)	73.4 (15.3)	73.8 (16.2)
RR before the SCT (breaths/min)	18.7 (6.6)	19.1 (6.7)	18.2 (6.5)
RR after the SCT (breaths/min)	29.0 (7.9)	28.9 (5.8)	29.2 (9.8)
Δ RR (breaths/min)	10.3 (6.4)	9.8 (6.2)	11.0 (6.7)

Data are shown with mean and standard deviation (SD) or N and Percentage of total (%).

**Table 2:** SCTt/12 m (s) centiles distribution by age and sex based on 827 test runs of children and adolescents aged 9–17 years.

Sex	Age (Years)	n	5	25	50	75	95
Girls	9	17	41.3	45.4	49.3	53.8	
	10	32	37.9	43.2	47.2	50.6	53.4
	11	47	39.3	41.7	45.3	48.6	55.5
	12	64	38.8	41.5	44.6	47.8	53.4
	13	38	38.4	41.1	43.6	45.8	52.0
	14	73	38.0	41.5	43.8	46.4	53.8
	15	48	38.7	42.1	44.3	46.8	53.8
	16	43	33.7	36.2	38.4	41.8	48.0
	17	17	39.8	41.8	42.7	43.9	
Boys	9	21	37.3	41.6	43.5	50.5	56.1
	10	29	38.7	41.4	43.7	46.8	57.5
	11	69	37.1	40.1	42.3	45.0	49.4
	12	68	35.5	39.3	42.7	45.0	50.7
	13	58	35.9	38.7	41.9	44.3	49.2
	14	61	35.1	38.1	40.8	43.6	50.8
	15	71	34.0	37.0	39.6	43.2	45.0
	16	50	33.7	36.2	38.4	41.8	48.0
	17	21	32.7	37.6	38.9	42.7	47.2

Data are shown in percentiles(s) or N.

## Data Availability

Data cannot be shared publicly because participants did not provide explicit consent for data sharing in accordance with the European Union’s General Data Protection Regulation and relevant German privacy laws. However, data can be made available to researchers who meet the criteria for access to confidential information through the Research Ethics Board of Ludwig-Maximilians-Universität Munich, Germany. Requests should be directed to: ethikkommission@med.uni-muenchen.de.

## Abbreviations

The following abbreviations are used in this manuscript:

BMI	Body Mass Index
BP	Blood pressure
bpm	Beats per minute
CPET	Cardiopulmonary Exercise Testing
CRF	Cardiorespiratory Fitness
CVD	Cardiovascular diseases
HR	Heart Rate
PEF	Peak Expiratory Flow
PA	Physical activity
RR	Respiratory Rate
SCT	Stair Climbing Test
SCTt	Stair Climbing Test time
SCTt/12 m	Stair Climbing Test time normalized over 12 m
VO_2_max	Maximal Oxygen Uptake
6MWT	6 Minutes Walking Test
